# Comparative Evaluation of Digitization of Diagnostic Dental Cast (Plaster) Models Using Different Scanning Technologies

**DOI:** 10.3390/dj8030079

**Published:** 2020-08-02

**Authors:** Aalaa Emara, Neha Sharma, Florian S. Halbeisen, Bilal Msallem, Florian M. Thieringer

**Affiliations:** 1 Department of Oral and Maxillofacial Surgery, Faculty of Dentistry, Cairo University, Cairo 12613, Egypt; aalaa.emara@dentistry.cu.edu.eg; 2 Department of Oral and Cranio-Maxillofacial Surgery, University Hospital Basel, 4031 Basel, Switzerland; neha.sharma@unibas.ch (N.S.); ft@swiss-mam.ch (F.M.T.); 3 Medical Additive Manufacturing Research Group (MAM), Department of Biomedical Engineering, University of Basel, 4123 Allschwil, Switzerland; 4 Basel Institute for Clinical Epidemiology and Biostatistics, Department of Clinical Research, University Hospital Basel, University of Basel, 4031 Basel, Switzerland; floriansamuel.halbeisen@usb.ch

**Keywords:** accuracy, CBCT, dental cast model, optical scanner, intraoral scanner

## Abstract

Rapidly developing digital dental technologies have substantially simplified the documentation of plaster dental models. The large variety of available scanners with varying degrees of accuracy and cost, however, makes the purchase decision difficult. This study assessed the digitization accuracy of a cone-beam computed tomography (CBCT) and an intraoral scanner (IOS), as compared to a desktop optical scanner (OS). Ten plaster dental models were digitized three times (n = 30) with each scanner. The generated STL files were cross-compared, and the RMS values were calculated. Conclusions were drawn about the accuracy with respect to precision and trueness levels. The precision of the CBCT scanner was similar to the desktop OS reference, which both had a median deviation of 0.04 mm. The IOS had statistically significantly higher deviation compared to the reference OS, with a median deviation of 0.18 mm. The trueness values of the CBCT was also better than that of IOS—median differences of 0.14 and 0.17 mm, respectively. We conclude that the tested CBCT scanner is a highly accurate and user-friendly scanner for model digitization, and therefore a valuable alternative to the OS. The tested IOS was generally of lower accuracy, but it can still be used for plaster dental model digitization.

## 1. Introduction

The advancement in computer-aided design/computer-aided manufacturing (CAD/CAM) technologies has significantly influenced the innovations in digital dentistry [1]. Digital dental technology (DDT) provides more accurate solutions and is applied in prosthetic-driven implant surgeries, orthodontics, and treatment planning [2,3,4]. To combat the issues with dental model storage, digitization processes are often beneficial to generate prompt three-dimensional (3D) virtual datasets. The virtual models not only offer smooth communication of digital data among clinicians, but also allow for more a comfortable documentation of patient records [5,6].

Documentation of plaster dental models is usually achieved by scanning the models that have been created on the basis of intraoral impressions. Dental digitization methods have continued to improve and are based on surface contact or non-contact technologies [7,8]. Intra- and extra-oral methods have been used to acquire 3D images of dental models. Currently, the most accurate digitization of plaster dental models is achieved through optical-based desktop scanner systems. An optical scanner (OS) is an extra-oral digitization method that uses a white light that is cast on the plaster dental model. Later, using a high-resolution camera, the projected pattern is captured, and the creation of a 3D image of the model is accomplished. Dental labs often prefer optical digitizers, as these involve less acquisition time for scan construction [9,10].

An alternative to OS for the digitization procedures of plaster dental models is an intraoral scanner (IOS). Numerous studies have assessed the accuracy of IOS for dental implantology and fixed prosthodontics [11,12,13]. According to ISO 5725-1 guidelines, accuracy comprises of two components, i.e., trueness and precision. The trueness signifies the variations amongst the test and the original images/scans. On the other hand, the precision is the variation amid the images from the same scanning system [14]. According to Flugge et al., virtual models generated by extra-oral digitization with iTero IOS devices were of acceptable accuracy and qualified for use in appliance manufacturing and treatment planning [15].

Another emerging and very promising technology is the cone-beam computed tomography (CBCT). Further advancements in the CBCT systems have made the digitization of plaster dental models possible [16]. Several CBCT manufacturers have started integrating extra cast digitization tools into their machines to simplify the workflow for data acquisition and surface extraction. Due to the various advantages of dental model digitization procedures, the prospective use of these technologies must be weighed against their accuracy and costs. For a dental practitioner who already has a CBCT or an IOS device, the procurement of another digitization system might seem unnecessary.

Therefore, this in-vitro study aimed to evaluate whether the CBCT and IOS are acceptable digitization technologies for dental models, commonly employed for diagnostic data storage as well as digital planning purposes. Furthermore, we assessed whether the accuracy of the virtual dental models generated by CBCT and IOS technologies were comparable with the ones generated by OS devices.

## 2. Materials and Methods

### 2.1. Experimental Models

Five pairs of maxillary and mandibular plaster dental models were randomly chosen from the archived model database of patients treated at the Department of Oral and Cranio-Maxillofacial Surgery of the University Hospital Basel, Switzerland. Selection was based upon the following inclusion criterion: (1) Adult dental cast models with complete dentition and/or partially edentulous regions (Table 1). Models with the following features were excluded: (1) models with hypodontia or supernumerary teeth; (2) models with fractured teeth; (3) models with retainers, appliances, or prosthesis; (4) models with voids, cracks, and/or visible porosities.

### 2.2. Scanning Devices and Strategies

The digitization systems included in this study were: a desktop OS device (IScan L series LI70910, Imetric 3D SA, Courgenay, Switzerland), a CBCT scanning device (CS 9000 3D, Carestream Dental LLC, Atlanta, GA, USA), and an IOS device (Trios 3, 3Shape A/S Dental Systems, Copenhagen, Denmark). The scanning procedures were conducted at a room temperature of 21 °C, with a relative humidity of 55 ± 3%. A single operator (A.E.), proficient in operating the scanners, performed all the image acquisition procedures in the following pre-determined order:

First, the plaster dental models were digitized in the OS device. This scanner uses an automatic triangulation function to create a 3D surface of the dental cast. The reliability of the OS (as a reference scanner for this study) was assessed, and each of the ten plaster dental models was digitized three times (n = 30). All virtual models were saved in a Standard Tessellation Language (STL) file format for further analysis. Next, the dental models were scanned with the CBCT imaging system. The CBCT acquisition technical parameters were—resolution: 0.076 mm, FOV: 0.050 × 0.037 mm, exposure time ~12 s, isotropic voxel size: 0.076 mm. Each plaster dental model was scanned three times (n = 30) and saved in the STL file format. Lastly, the plaster dental models were digitized with an IOS device. The scanning protocol was based on the manufacturer’s instructions and was as follows: each arch’s occlusal surface was scanned with a smooth movement, starting at the last tooth on the left. The scanner head was then turned 45° to scan the palatal surfaces. Then, the scanner head was turned 45° in the opposite direction to scan the buccal surfaces. Each dental cast model was digitized three times (n = 30). During the IOS scanning process, three distorted images with missing data points were produced, and hence, these scans were repeated to avoid potential discrepancies. Finally, the datasets were saved in the STL file format.

### 2.3. Image Registration and Accuracy Analysis

**Figure 1:**
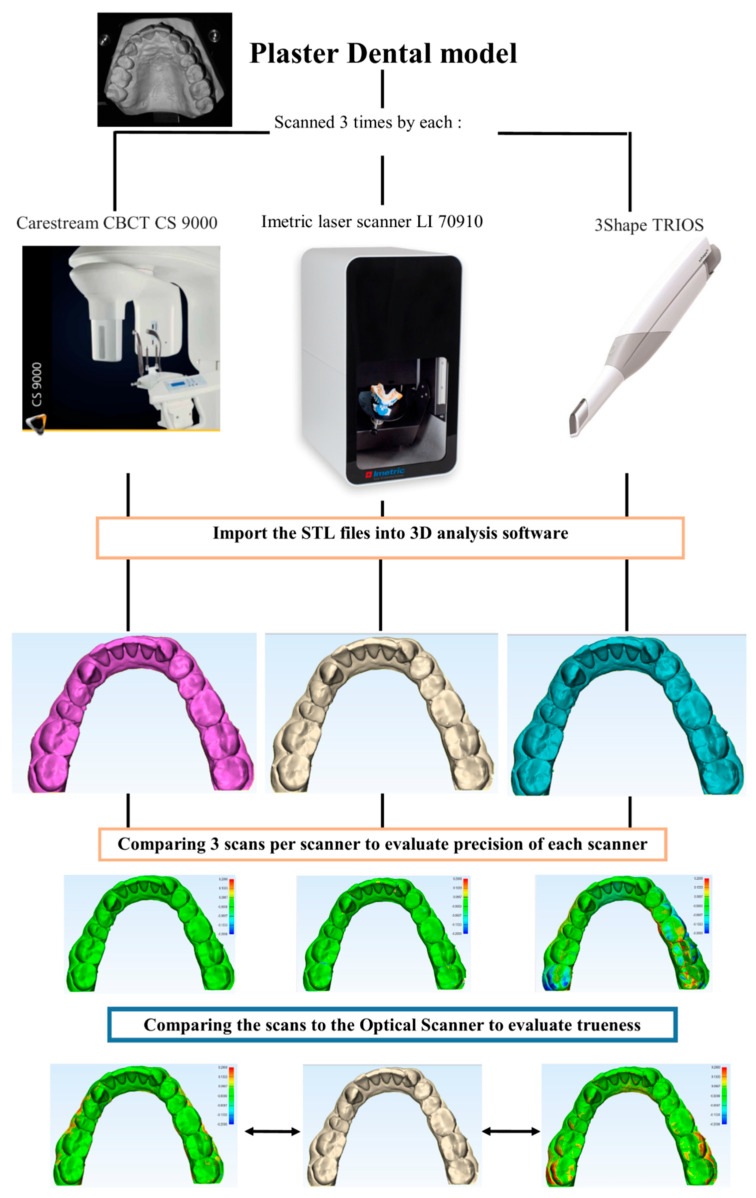
Overview of the protocol for accuracy assessment (trueness and precision) of scanners. Imetric laser scanner (Artec3D^®^), 3D digital intraoral scanner (Trios^®^), CBCT CS9000 scanner (Carestream^®^).

The STL files of the OS (reference), the CBCT, and the IOS were imported into a 3D part-comparison analysis software program (3-matic medical, version 13.0, Materialise NV, Leuven, Belgium). All non-essential data, such as soft tissue and areas outside the gingivobuccal regions, were digitally trimmed using identical cutting planes. Next, point-based surface registration using a best-fit alignment method was carried out, and accurate superimposition of CBCT and IOS datasets with OS was achieved. Then, a part-comparison analysis was performed, and color histograms were generated to visualize the deviations. For the quantitative assessment of deviations, the software computed the root mean square (RMS) values. These values represented the absolute deviations between the two datasets. The three optical scans acquired from each case were cross-compared to evaluate the precision of each scanning system. Next, the first optical scan from each case was used as the reference for the trueness assessment of the CBCT and IOS scanning devices (Figure 1). The value of RMS was calculated automatically in the software by using the following formula:

$$\mathrm{RMS}=\sqrt{\frac{1}{2}\overset{n}{\underset{i=1}{\sum }}x_{i}^{2}},$$

If point x in the reference STL file has the closest point x’ in the entity file, then X_n_ is the distance between x and x’, and n is the total number of point pairs in both digitized files.

### 2.4. Statistical Analysis

Descriptive statistics were used, and data were represented as mean, standard deviation (SD), median, and first- and third quartile ranges (interquartile range; IQR). RMS values were computed for each scanner to summarize the quantitative characteristics and to assess the accuracy of the scanners. A Shapiro–Wilk test was used to attest to the normal distribution of the data. For intergroup differences between scanners, a one-way analysis of variance (ANOVA) with Tukey Kramer post-hoc pairwise tests or a Kruskal–Wallis test with pairwise Wilcoxon rank-sum (Mann–Whitney U) post-hoc tests, adjusted for multiple-testing using a Holm-Bonferroni correction, was applied. The statistical analysis was done in R software (R Core Team 3.4.1, The R Foundation for Statistical Computing). The significance level was set α = 0.05.

## 3. Results

### 3.1. Precision Assessment

The overall precision assessment results for all the scanners are summarized in Table 2. For the OS, the precision RMS values resulted in a mean (SD) value of 0.06 (0.08) mm, along with a median (IQR) value of 0.04 (0.03–0.05) mm. IOS precision RMS values had a mean (SD) value of 0.23 (0.18) mm, with a median (IQR) value of 0.18 (0.1–0.31) mm. For the CBCT scanner, the RMS values showed a mean (SD) of 0.05 (0.03) mm, and a median (IQR) value of 0.04 (0.03–0.07) mm.

**Figure 2:**
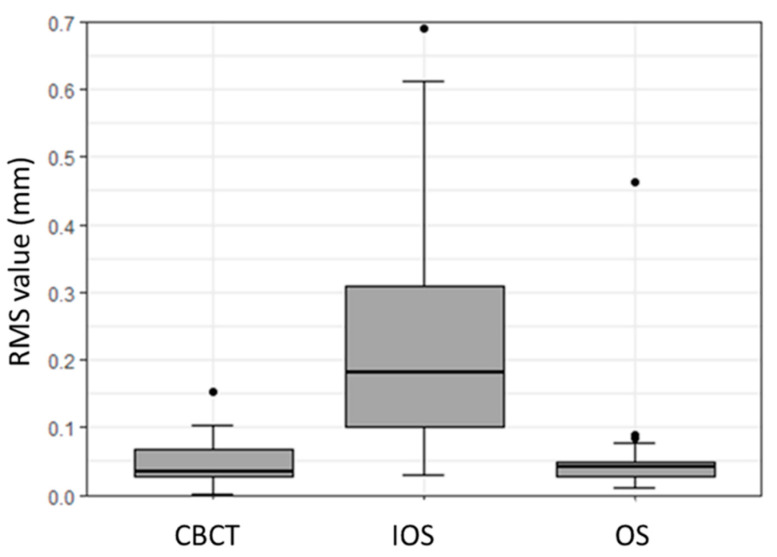
Box plot illustrating precision assessment concerning root mean square (RMS) values (mm) for CBCT, IOS, and OS.

The subsequent box plot graph denotes the quantitative deviation for the precision of each scanner (Figure 2). The IOS displayed a statistically significant difference in terms of precision when compared with the OS and the CBCT scanners (*p* < 0.01). In contrast, no statistically significant difference was noticed in the precision of the OS and the CBCT scanners (*p* < 0.79).

**Figure 3:**
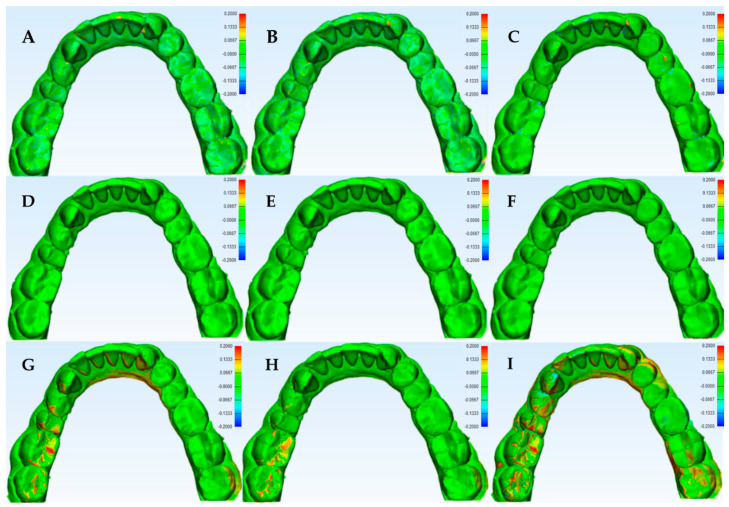
3D color histogram for precision analysis of the tested scanners in one model. (**A**–**C**) are 1st vs. 2nd, 2nd vs. 3rd, 1st vs. 3rd scans. The first row (**A**–**C**) for OS, (**D**–**F**) for CBCT and (**G**–**I**) for the IOS.

A 3D color histogram was used for each scanner to point out areas of higher deviation. The mandibular model scans generally had higher deviations than the maxillary ones. The precision color histogram analysis is shown in Figure 3.

**Figure 4:**
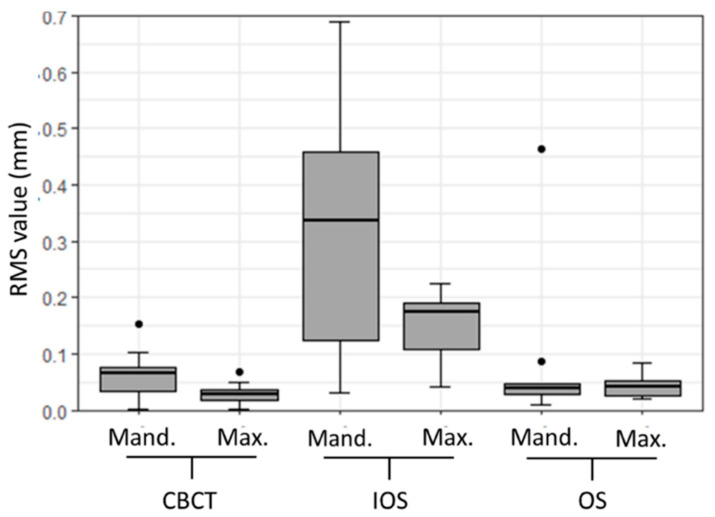
Box plot illustrating precision root mean square (RMS) values (mm) in the maxillary (Max.) and mandibular (Mand.) plaster dental models for CBCT, IOS, and OS.

Additionally, we performed a quantitative assessment of the scanners’ precision with respect to location, i.e., separately for maxillary and mandibular plaster models. The results are shown in Table 3 and Figure 4. The precisions of the maxillary and mandibular models performed with the IOS proved to be statistically significantly different from the models performed with the other two scanners (*p* < 0.01).

### 3.2. Trueness Assessment

**Figure 5:**
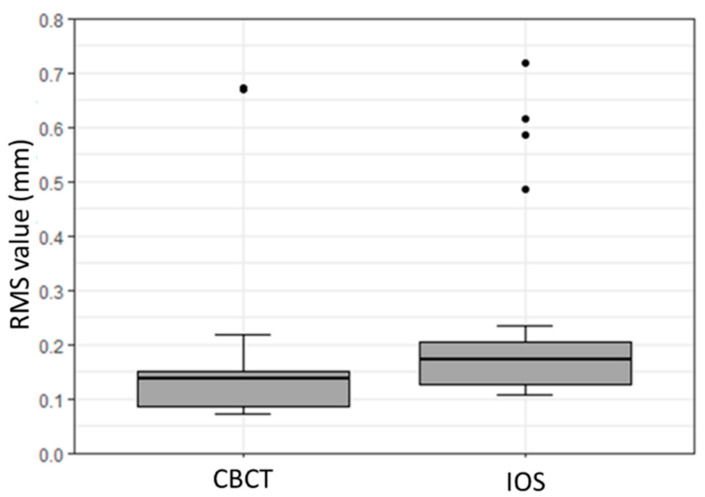
Box plot showing the overall trueness assessment concerning root mean square (RMS) values (mm) for CBCT and IOS.

**Figure 6:**
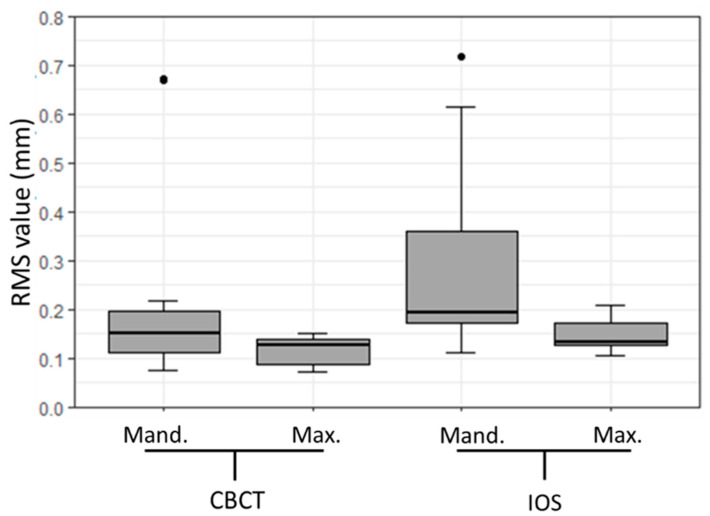
Box plot illustrating trueness root mean square (RMS) values (mm) in the maxillary (Max.) and mandibular (Mand.) dental plaster models for CBCT and IOS.

A quantitative deviation distribution for trueness assessment for CBCT and IOS was performed with OS as reference. For the CBCT, the comparison analysis displayed a median (IQR) difference of 0.14 (0.09–0.15) mm, with a range of 0.07 to 0.67 mm. On the contrary, the IOS revealed a median (IQR) difference of 0.17 (0.13–0.2) mm, with a range of 0.11 to 0.72 mm. The overall trueness results of CBCT and IOS scanners, when compared to the OS and with respect to location, i.e., maxillary and mandibular plaster dental models, are depicted in Figure 5 and Figure 6.

**Figure 7:**
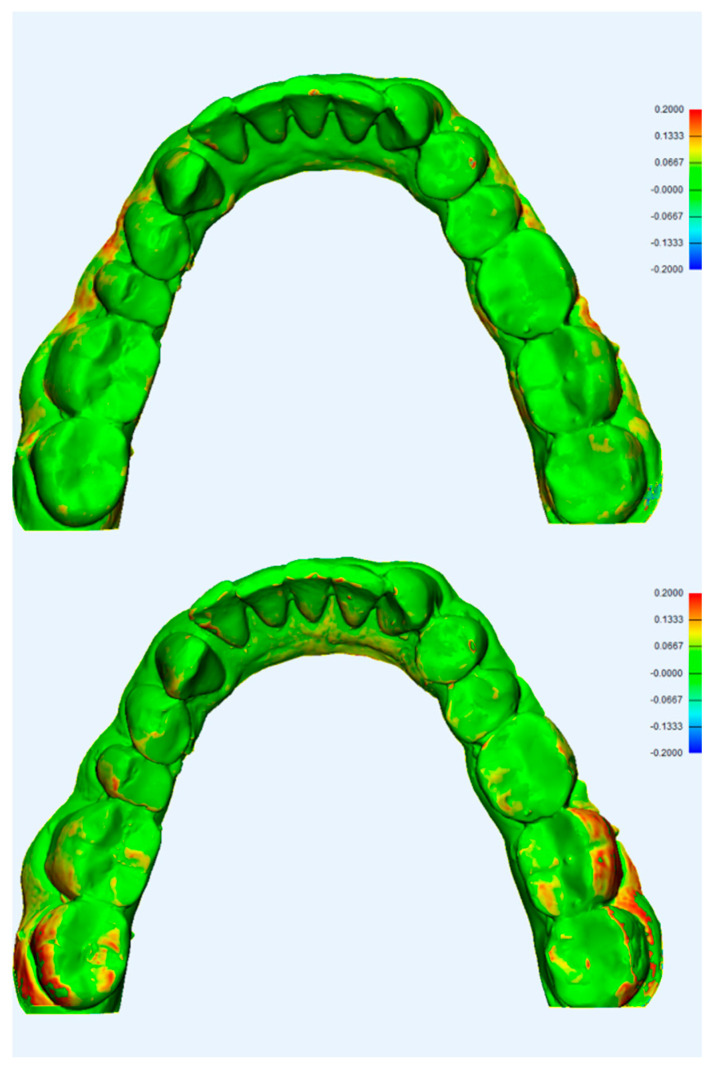
3D color histogram showing trueness assessment of CBCT scan (**top**) and IO scan (**bottom**), note the higher deviation scores in the posterior regions.

A color histogram also shows differences in trueness between CBCT and IOS, with higher deviation values noted at the incisal/occlusal anatomy of the teeth and the posterior region, as seen in Figure 7.

## 4. Discussion

With the incorporation of digital workflows into dentistry, the acquisition of accurate virtual 3D models has become essential. Digital workflows entail higher efficiency and facilitate data storage, reproducibility, and treatment documentation, and can even lead to advanced treatment concepts. The continuous introduction of new equipment with technological updates in the market, however, has made the selection of an appropriate tool difficult for clinicians. Therefore, this study aimed to provide evidence on accuracy (precision and trueness) of commonly used CBCT and IOS devices, as validated against an OS, to digitize plaster dental models.

The results from this study suggest that the OS (reference scanner) is a highly reliable digitization tool, displaying no statistically significant differences amongst the scans. The consistency in generated scan datasets was assessed by applying a best-fit alignment software protocol, as reported by Mangano et al. [17]. Moreover, a significant difference in terms of trueness of scans produced with the IOS and the CBCT was noticed; this difference was greater (with higher deviations) in the mandibular models than in the maxillary models. In agreement with several studies reported in the literature [18,19,20,21,22,23], our results confirm that the precision of OS was within an acceptable range. Even if these studies were able to prove the reliability of the OS for the digitalization of dental models, other reports have outlined its limitation in cases with improper dental alignment [21]. Our results support this, as we found the most considerable deviations in the 3D color histogram in areas with abnormal dental alignment, such as a rotated tooth, buccally/palatally inclined teeth, or edentulous spaces. Apart from this finding, the optical scanner was highly precise and qualified as a reference, as stated in other literature reports [17,24,25]. When the scanned object is placed onto a platform (rather than handheld), the OS seems to be a simple and straightforward procedure; in our study, none of the models required a second scan due to missing data or non-completion of the scan. However, despite all the advantages, it is very cost-intensive and therefore unaffordable for many dental labs.

The second scanner evaluated in this study was the CS 9000 3D CBCT. Trueness assessment showed a median (IQR) difference of 0.14 (0.09–0.15) mm in comparison to the reference OS. This is considered to lie within a clinically acceptable margin of error, and should therefore not affect the clinical applications of this digitalization process. Our results are similar to the findings reported by Becker et al., who stated that, even if the scanner’s precision is lower than that of the reference desktop scanner, it is still clinically acceptable [26]. In our study, the precision of the CBCT scanner was found to be slightly higher (statistically insignificant difference) than the reference OS (mean of 0.05 ± 0.03 vs. 0.06 ± 0.08 mm), with the median ranges in slight favor of the OS. This may be attributed to the model digitization protocol provided by the Carestream products. The digitization protocol includes a quick primary scan (with the model in its proper position, which is pre-set according to the type of the model and average arch size) to ensure the appropriate focal position and to maintain the optimal accuracy of the scan. This is not the case in an OS, where the cast is placed on the fixed scanning platform, and the scan starts immediately. However, the digitization process in Carestream was very simple and straightforward, similar to the findings reported by Becker et al. in their study. Additionally, these digitization tools increase the scanning accuracy, which supports the result that the precision of Carestream scans were superior to other types of CBCT scans [26].

Our results concerning the analysis of CBCT scans are also in line with those reported in the literature, not as much for scanning models but for scanning dental impressions [1]. Park et al. reported the accuracy of CBCT in scanning impressions and compared it to measurements made on plaster dental models [27]. The average difference in this study was 0.15 mm, which was also considered clinically acceptable; this result coincides with the result of our study—a median difference of 0.14 mm. None of the scans acquired by the CBCT required repetition due to missing data or non-completion of the scans, and the handling of the machine was simple. Assessment of the scans showed that the deviations, whether in precision or trueness assessment, were mainly located on the posterior regions, particularly in the mandible models and at edentulous spaces. Higher deviations in the posterior regions is also in accordance with what Oh et al. reported in their study, even though they assessed the accuracy of scanners on a maxillary typodont rather than plaster dental models [23]. This may be explained by the narrower ridge present in this area, with prominent curvatures and complex dental anatomy.

The third assessed scanner was the 3Shape TRIOS IOS. This handheld scanner is passed along the different surfaces of the dental model to thoroughly scan it according to a pre-determined protocol. The software then calculates the scanned areas to generate a virtual model. If the reconstruction of data points cannot be generated, an error message appears that prompts the repetition of the scanning procedure. A repeated scan was needed in three of our models (all mandibular). Moreover, it took some time for the assessor to get used to the procedure, as it is quite sensitive; the assessor started with the last tooth on the left side, moving all over the dental arch, scanning the occlusal surfaces, then the buccal and lingual/palatal surfaces. The presence of missing data, artifacts, or distorted images in intraoral scanning procedures has been reported in the literature before [28,29].

Intraoral scanners have what is called a focal depth, which is the optimal distance at which the target to be scanned should be positioned to acquire an ideal virtual model. In the case of natural randomly selected models, malalignment of teeth and the presence of edentulous spaces in some of the studied cases meant that some areas would move closer or further away from the focal depth, which makes the scan inaccurate [30]. This was similar in a review article on different intraoral scanners and may influence the results [31]. Moreover, controlling the handheld scanner to keep the appropriate focal depth throughout the scanning procedure is challenging. The more recent version of the IOS that was used for our study tries to overcome this issue by adding a handle to the scanner, which was simply pen-shaped in its earlier versions. Lighting conditions also influence the accuracy of the IOS, which were reported to be different depending on the IOS manufacturer [32]. As reported in that study, room lighting, as used in our study, is ideal for the TRIOS IOS.

The trueness of the IOS, when compared to the reference scanner, revealed a median (IQR) difference of 0.17 (0.13–0.2) mm, with a range of 0.11 to 0.72 mm; this means—as expected, and similar to what was reported in other studies [15,33]—that the IOS is associated with a lower trueness than the CBCT scanner. Moreover, the precision of the IOS was the lowest among the studied scanners, with a mean (SD) deviation of 0.23 (0.18) mm and a median (IQR) deviation of 0.18 (0.1–0.31) mm. This is also in line with literature reports; the authors of these reports still consider this an acceptable margin of deviation, and recommend the use of this scanner for clinical applications [17,19,24,25,34,35,36,37,38]. The higher deviations reported in our mandibular models are not in agreement with what was reported in the majority of the published studies, in which maxillary models showed higher deviations. This may be attributed to the narrower anatomy and complex dental alignment of the mandibular models that were included in this study, which made remaining at the optimal focal depth during scanning difficult. An important confounding factor in this study is that the handling and digitization of the models were dependent on the clinician, especially when using the IOS wand, which may have led to an operator bias. The limited number of plaster dental models used in this study and the lack of detailed assessment of the accuracies in edentulous/implant/malaligned teeth are further limitations and should be reassessed in further studies.

Although a consensus on the definition of clinically acceptable deviation is currently lacking and is left to the clinicians’ discretion, some authors suggest it to range from 0.15 to 0.2 mm [24,34]. According to this range, we may state that the scanners tested in this study can be used for model digitization with acceptable accuracy. The tested CBCT scanner showed high precision and trueness. To avoid additional procurement costs to the clinicians, those who already have a CBCT device with a scanning protocol need not purchase an OS for digitization of the models. On the other hand, despite the results of the IOS, the deviations still lie within the clinically acceptable range, qualifying for a tool for the digitization of plaster dental models. Being the least expensive option of the tested devices, clinicians should assess their needs, such as digitization, pre-prosthetic assessment, orthodontic evaluation, and so on.

## 5. Conclusions

We conclude that the OS is the best option for the digitization of dental models. The CBCT, however, proved to be a highly precise alternative. Even if the tested IOS showed the lowest results in terms of accuracy, it is still a valid affordable option for model digitization, with results falling within the “clinically acceptable” range.

## Figures and Tables

**Table 1:** Classification of plaster dental models used for scanning.

	Kennedy Classification	
Group	Maxillary Model	Mandibular Model
Case 1	Not applicable	Class III
Case 2	Class III	Not applicable
Case 3	Not applicable	Not applicable
Case 4	Not applicable	Class III
Case 5	Class III	Not applicable

**Table 2:** Overall results for precision RMS values (mm) in different scanners.

Scanner	Mean (SD)	Median (Q1 to Q3)	Range
OS	0.06 (0.08)	0.04 (0.03 to 0.05)	0.01 to 0.46
IOS	0.23 (0.18)	0.18 (0.1 to 0.31)	0.03 to 0.69
CBCT	0.05 (0.03)	0.04 (0.03 to 0.07)	0.002 to 0.15

**Table 3:** The statistical outcome of a precision comparison between different scanners with respect to maxillary/mandibular dental cast models.

	CBCT (max.)	CBCT (mand.)	OS (max.)	OS (mand.)	IOS (max.)
CBCT (mand.)	0.03				
OS (max.)	0.378	0.376			
OS (mand.)	0.411	0.376	0.862		
IOS (max.)	<0.01 *	<0.01 *	<0.01 *	<0.01 *	
IOS (mand.)	<0.01 *	<0.01 *	<0.01 *	<0.01 *	0.376

* *p* < 0.05 represents a statistically significant difference.

## Abbreviations

**ANOVA**	Analysis of variance
**CAD/CAM**	Computer-aided design/computer-aided manufacturing
**CBCT**	Cone-beam computed tomography
**DDT**	Digital dental technology
**IOS**	Intraoral scanner
**OS**	Optical scanner
**STL**	Standard tessellation language
**RMS**	Root mean square
**3D**	Three-dimensional
